# Prizes on Tuberculosis

**Published:** 1908-04

**Authors:** 


### Prizes on Tuberculosis

THE CENTRAL COMMITTEE OF THE INTERNATIONAL CONGRESS ON TUBERCULOSIS HAS ANNOUNCED THE OFFER OF THE FOLLOWING PRIZES:

I. A prize of $1 000.00 is offered for the best evidence of effective work in the prevention or relief of tuberculosis by any voluntary association since the last International Congress in 1905. In addition to the prize of $1,000.00, two gold medals and three silver medals will be awarded. The prize and medals will be accompanied by diplomas or certificates of award.

Evidence is to include all forms of printed matter, educational leaflets, etc.; report showing increase of membership, organisation, classes reached—such as labor unions, schools, churches, etc.; lectures given; influence in stimulating local boards of health, schools, dispensaries, hospitals for the care of tuberculosis; newspaper clippings of meetings held; methods of raising money; method of keeping accounts.

Each competitor must present a brief or report in printed form. No formal announcement of intention to compete is required.

II. A prize of $1,000.00 is offered for the best exhibit of an existing sanatorium for the treatment of curable cases of tuberculosis among the working classes. In addition to the prize of $1,000,00, two gold medals and three silver medals will be awarded. The prize and medals will be accompanied by diplomas or certificates of award.

The exhibit must show in detail construction, equipment, management, and results obtained. Each competitor must present a brief or report in printed form.

III. A prize of $1,000.00 is offered for the best exhibit of a furnished house, for a family or group of families of the working class, designed in the interest of the crusade against tuberculosis. In addition to the prize of $1,000.00, two gold medals and three silver medals will be awarded. The prize and medals will be accompanied by diplomas or certificates of award. This prize is designed to stimulate efforts towards securing a maximum of sun-light ventilation, proper heating, and general sanitary arrangement for an inexpensive home. A model of house and furnishing is required. Each competitor must present a brief with drawings, specifications, estimates, etc., with an explanation of points of special excellence. Entry may be made under competitor's own name.

IV. A prize of $1,000.00 is offered for the best exhibit of a dispensary or kindred institution for the treatment of the tuberculous poor. In addition to the prize of $1,000.00, two gold medals and three silver medals will be awarded. The prize and medals will be accompanied by diplomas or certificates of award.

The exhibit must show in detail construction, equipment, management, and results obtained. Each competitor must present a brief or report in printed form.

V. A prize of $1,000.00 is offered for the best exhibit of a hospital for the treatment of advanced pulmonary tuberculosis. In addition to the prize of $1,000.00, two gold medals and three silver medals will be awarded. The prize and medals will be accompanied by diplomas or certificates of award.

The exhibit must show in detail construction, equipment, management and results obtained. Each competitor must present a brief or report in printed form.

VI. The Hodgkins Fund Prize of $1,500.00 is offered by the Smithsonian Institution for the best treatise that may be submitted on "The Relation of Atmospheric Air to Tuberculosis."

The detailed definition of this prize may be obtained from the Secretary-General of the International Congress or Secretary of the Smithsonian Institution, Chas. D. Walcott.

VII. Prizes for Educational Leaflets:

A prize of $100.00 is offered for the best educational leaflet submitted in each of the seven classes defined below. In addition to the prize of $100.00, a gold medal and two silver medals will be awarded in each class. Each prize and medal will be accompanied by a diploma or certificate of award.

Competitors must be entered under assumed names.

A. For adults generally (not to exceed 1 000 words).

B. For teachers (not to exceed 2,000 words).

C. For mothers (not to exceed 1,000 words).

D. For in-door workers (not to exceed 1,000 words).

E. For dairy farmers (not to exceed 1,000 words).

F. For school children in grammer school grades (not to exceed 500 words).

In classes A, B, C, D, E, and F, brevity of statement without sacrifice of clearness will be of weight in awarding. All leaflets entered must be printed in the form they are designed to take.

G. Pictorial booklet for school children in primary grades and for the nursery.

Class G. is designed to produce an artistic picture-book for children, extolling the value of fresh air, sun-light, cleanliness, etc., and showing contrasting conditions. "Slovenly Peter" has been suggested as a possible type. Entry may be made in the form of original designs without printing.

VIII. A gold medal and two silver medals are offered for the best exhibits sent in by any States of the United States, illustrating effective organisation for the restriction of tuberculosis. Each medal will be accompanied by a diploma or certificate of award.

IX. A gold medal and two silver medals are offered for the best exhibits sent in by any State or Country (the United State excluded), illustrating effective organisation for the restriction of tuberculosis. Each medal will be accompanied by a diploma or certificate of award.

X. A gold medal and two silver medals are offered for each of the following exhibits; each medal will be accompanied by a diploma or certificate of award; wherever possible each competitor is required to file a brief or printed report:

A. For the best contribution to the pathological exhibit.

B. For the best exhibit of laws and ordinances in force June 1, 1908, for the prevention of tuberculosis by any State of the United States. Brief required.

C. For the best exhibit of laws and ordinances in force June 1, 1908, for the prevention of tuberculosis by any State or Country (the United States excluded). Brief required.

D. For the best exhibit of laws and ordinances in force June 1, 1908, for the prevention of tuberculosis by any municipality in the world. Brief required.

E. For the society engaged in the crusade against tuberculosis having the largest membership in relation to population. Brief required.

F. For the plans which have been proven best for raising money for the crusade against tuberculosis. Brief required.

G. For the best exhibit of a passenger railway car in the interest of the crusade against tuberculosis. Brief required.

H. For the best plans for employment for arrested cases of tuberculosis. Brief required.

XI. Prizes of two gold medals and three silver medals will be awarded for the best exhibit of a work-shop or factory in the interest of the crusade against tuberculosis. These medals will be accompanied by diplomas or certificates of award.

The exhibit must show in detail construction, equipment, management, and results obtained. Each competitor must present a brief or report in printed form.

The following constitute the Committee on Prizes:

DR. CHARLES J. HATFIELD, Philadelphia, *Chairman*
DR. THOMAS G. ASHTON, Philadelphia, *Secretary*
DR. EDWARD R. BALDWIN, Saranac Lake

DR. SHERMAN G. BONNEY, Denver
DR. JOHN L. DAWSON, Charleston, S. C.
DR. H. B. FAVILL, Chicago
DR. JOHN B. HAWES, 2D., Boston
DR. H. D. HOLTON, Brattleboro
DR. E. C. Levy, Richmond, Virginia
DR. CHARLES L. MINOR, Ashville, N. C.
DR. ESTES NICHOLS, Augusta, Me.
DR. M. J. ROSENAU, Washington
DR. J. MADISON TAYLOR, Philadelphia
DR. WILLIAM S. THAYER, Baltimore
DR. LOUIS M. WARFIELD, St. Louis

